# Understanding the Relationship Between Loneliness and Sleep, and Their Influence on Mental Health of a High-Adversity-Exposed School Sample of Kenyan Adolescents

**DOI:** 10.5334/aogh.4810

**Published:** 2025-08-14

**Authors:** Manasi Kumar, Shillah Mwaniga Mwavua, Sabrina Cheng, Alicia Chung, Leonard Njeru Njiru, Georgina Obonyo, Mohammad Dayow, Keng-Yen Huang

**Affiliations:** 1 Institute for Excellence in Health Equity, New York University School of Medicine, 180 Madison Avenue, 7th Floor, New York, NY, 10016, USA; 2 Nairobi City County Health Wellness and Nutrition department, Nairobi, Kenya; 3 Faculty of Behavior and Movement Sciences, Department of Clinical, Neuro Developmental Psychology, Vrije University Amsterdam, The Netherlands; 4 Department of Population Health, New York University School of Medicine, 180 Madison Avenue, 6th Floor, New York, NY, 10016, USA; 5 Specialist psychiatrist, Ministry of Health, Muranga County, P.O. Box 26732-00100, Nairobi, Kenya; 6 mSELY study coordinator, Café Tabasamu, Nairobi, Kenya; 7 Head, School Health Program, County Health Wellness and Nutrition, Nairobi City Hall, City Hall way, P.O. Box 30075-00100, Nairobi, Kenya

**Keywords:** adolescents, kenya, loneliness, sleep, mental disorders, adversities, school, family

## Abstract

*Background:* Loneliness is emerging as a key risk factor for child and adolescent mental health. Exacerbated by lack of support, busy routines, continuous adversities, and poor social networks, it is a public health concern. Sleep is essential for healthy development and emotional regulation, critical for modulating risk‑taking, and determines optimal learning and mental health. However, the connection between loneliness and sleep and their impact on mental health and educational outcomes is not well known in low‑ and middle‑income countries (LMICs) like Kenya, where a large portion of the population is young.

*Objectives:* (1) Examine the bidirectional relationship between loneliness and impaired sleep in a Kenyan adolescent cohort. (2) assess the individual and joint contribution of loneliness and sleep impairment relationship in common mental health problems such as anxiety, depression, and anger, while controlling for adverse childhood experiences (ACEs).

*Methods:* A cross‑sectional study with 70 adolescents (ages 11–15) from Nairobi schools. Measures used: Loneliness (NIH Toolbox), Sleep Impairment (PROMIS), Mental Health (PROMIS‑Anger, Anxiety, Depression), ACEs (WHO ACEs‑IQ).

*Findings:* We found a strong association between loneliness and sleep impairment, even after controlling for ACEs (associated with 32.0%–33.9% of variance). Higher ACEs were also associated with increased loneliness and sleep impairment. Notably, after adjusting for the ACE confounder, both sleep impairment and loneliness were significantly associated with adolescent mental disturbances (anxiety, depression, and anger), with sleep impairment explaining greater variance (27.2%–30.4%) than loneliness (11.8%–27.4%) for the anxiety, anger, depression outcomes. Jointly, loneliness and sleep impairment were associated with 28.8%–30.4% of the variance in adolescents’ mental health problems.

*Conclusion:* Our article contributes new evidence that sleep health is critical to mental well‑being for adolescents living in high ACEs and LMIC contexts. Providing intervention strategies to reduce loneliness and promote sleep health should be considered to improve adolescent mental health.

## Introduction

Loneliness among adolescents is a growing public health burden with a global prevalence of 10.7%, and it, along with sleep disturbances, severely impacts adolescent health and development [1, 2]. These trends suggest that such issues are not only prevalent but may be worsening. Contributing factors include academic pressure, diminished family cohesion, and pervasive digital media use, which have been linked to rising rates of loneliness and sleep disturbances among adolescents worldwide. With socio‑economic stressors rampant in Sub‑Saharan African settings, children and adolescents are losing social protection networks rapidly that leads to a persistent sense of alienation and isolation. Despite national and global efforts [3, 4], a paucity of research evidence from LMICs creates evidence gaps. Adolescents in Kenya live in high‑adversity contexts, including enormous socio‑economic hardships, poor living conditions, and exposure to interpersonal violence [5]. Loneliness, a sub‑syndromal characteristic, can be exacerbated by these stressors and poor quality of life. These stressors, along with feelings of loneliness, can disrupt sleep. Sleep impairment can, in turn, trigger feelings of isolation, creating a vicious cycle. Loneliness is negatively related to happiness [6] such that loneliness and sleep problems together aggravate mental ill‑health.

Funded by the US Fogarty International Center and National Institutes of Health (5R21MH124149‑02), this study investigates the associations between sleep impairment, loneliness, and mental health outcomes in a sample of Kenyan adolescents with high exposure to adverse childhood experiences (ACEs), aiming to inform school‑based digital interventions for early detection and mental health promotion.

## Method

### Ethical approval, settings, participants, and tools

*Ethical approval* was sought from the AMREF Ethical review committee (P1122‑2021) and NACOSTI license was sought (NACOSTI/P/22/16992).

*Settings and Participants:* Adolescents aged 11–15 years were recruited from 5th–8th grades in Nairobi County schools. Assent was obtained from adolescents and consent from caregivers after explaining the study objective and resources for linkages on mental health services. Trained youth leaders conducted 90‑minute assessment sessions with the adolescents, following training in mental health assessments, ethics, and referral procedures for at‑risk adolescents.

## Measures

The NIH Toolbox‑Loneliness scale (7 items; α = .90 using our Kenya study sample) was used to assess loneliness symptoms. Sleep regulation was measured using the PROMIS‑Sleep Impairment Scale (4 items; α = .81 using our Kenya study sample). Mental health outcomes were assessed using PROMIS‑Anger (α = .82), Anxiety (α = .87), and Depression (α = .89). All the PROMIS measures were rated on a 5‑point scale (1 = never to 5 = Almost always).

*Confounder:* A 14‑item adapted Adverse Childhood Experiences International Questionnaire (ACE‑IQ) assessing childhood adversities at home, such as child maltreatment and other family dysfunction, through a yes–no questionnaire. A summary score was created under two categories—family‑based ACEs (10 ACEs areas) and poverty‑associated ACEs (4 items).

### Data collection procedure

Data was collected in partnership with the Nairobi County School Health teams. Adolescents completed a tablet‑based survey during pre‑arranged sessions with trained youth leaders. For adolescents in distress, referrals were made by psychiatrists, clinical psychologists, or county school health team members.

### Data analysis

To examine the unique and joint contribution of loneliness and sleep problems to adolescent mental health problems, we carried out three sets of linear regression models. We modeled loneliness and sleep impairment as predictors separately and jointly, and carried out the analysis separately for each mental health outcome. All the analyses control for the confounder ACEs. Data was analyzed using SPSS v23.

## Findings

The mean age of our participants was 12.49 years and 61.4% were females. Our key findings are summarized below.

***Financial and adversity challenges:*** 47.1% had financial challenges (not enough to eat, difficulty paying school fees, no housing); 68.6% had home ACEs, and 77.1% had any of financial adversity or home ACEs.

***Mental health risk:*** 31.4% with anxiety, 31.4% with depressive symptoms, and 18.6% for anger, 28.6% for sleep impairment, 37.1% for loneliness. No age or gender differences were found on study predictors and outcome measures in our sample.

***ACEs:*** Significant positive associations between ACEs and sleep impairment (*p <.001*) and loneliness *(p =.002*) were found (Table 1). Higher ACEs were associated with higher sleep impairment and loneliness. Significant positive associations between ACEs and mental health (Table 2). Findings indicated that ACEs were a confounder in the association between sleep/loneliness and mental health. Thus, subsequent regression association analyses adjusted for ACEs.

**Table 1:** Association between loneliness and sleep impairment (controlled for ACEs).

	SLEEP IMPAIRMENT		LONELINESS	
***Model 1***	B (SE)	*p*	B (SE)	*p*
ACEs‑T	.59 (.15)	<.001	.80 (.25)	.002
R²	18.1%	<.001	13.2%	.002
***Model 2 (either predictor)***				
ACEs‑T	.28 (.13)	.030	.20 (.22)	.367
Loneliness	.38 (.06)	<.001	NA	NA
Sleep Impairment	NA	NA	1.02 (.16)	<.001
R²∆ (for the added predictor)	32.0%	<.001	33.9%	<.001
R²‑Total (for joint effect for the two predictors)	50.1%		47.1%	

*Note: Correlation between sleep impairment and loneliness was r = .68 (p < .001).*

**Table 2:** ACEs, loneliness and sleep impairment as predictors for common adolescent mental health problems.

	PROMIS‑ANXIETY		PROMIS‑DEPRESSION		PROMIS‑ANGER	
***Model 1***	B (SE)	*p*	B (SE)	*p*	B (SE)	*p*
ACEs‑T	.65 (.32)	.047	.83 (.31)	.009	.39 (.20)	.054
R²	5.7 %		9.7 %		5.3%	
***Model 2***						
ACEs‑T	.24 (.32)	.452	.28 (.28)	.307	.17 (.20)	.416
Loneliness	.51 (.14)	<.001	.68 (.13)	<.001	.28 (.09)	.003
R²∆ (Loneliness)	14.9%		27.4%		11.8%	
***Model 3***						
ACEs‑T	–.03 (.30)	.918	.30 (.31)	.327	–.05 (.18)	.797
Sleep Impairment	1.15 (.21)	<.001	.89 (.22)	<.001	.74 (.13)	<.001
R²∆ (Sleep Impairment)	28.3%		27.2%		30.4%	
***Model 4***						
ACEs‑T	–.05 (.30)	.859	.19 (.29)	.500	–.05 (.19)	.801
Loneliness	.12 (.17)	.492	**.56 (.16)**	**<.001**	–.002 (.10)	.983
Sleep Impairment	**1.03 (.28)**	**<.001**	.32 (.26)	.225	**.74 (.17)**	**<.001**
R²∆ (for Loneliness and Sleep Impairment joint effect)	28.8%		28.8%		30.4%	
**R²‑Total** (for all 3 predictors)	**34.5%**		**34.8%**		**35.8%**	

***Association between loneliness and sleep impairment:*** The regression model showed that these two variables were significantly associated, even after controlling for ACEs.

***Contributions of loneliness and sleep on Mental Health:*** Both sleep impairment and loneliness were significantly associated with adolescent mental disturbances (anxiety, depression, and anger). Loneliness was associated with 11.8%–27.4% of the variance in mental health problems, above and beyond the contribution of ACEs (5.3%–9.7%). While sleep impairment was associated with 27.2%–30.4% of the variance in mental health problems above and beyond the contribution of ACEs. In addition, sleep impairment was associated with a greater proportion of the variance (28.3%–30.4%) in anxiety and anger compared to loneliness (11.8%–14.9%). For depression, sleep impairment and loneliness accounted for similar proportions of the variance. Overall, loneliness and sleep impairment were associated with 28.8%–30.4% of the variance and ACEs, loneliness, and sleep impairment combined were associated with 34.5%–35.8% of the variance in mental health problems.

## Discussion

Our study, albeit a small sample from a peri‑urban school population in Nairobi, shows that ACEs such as abuse, exposure to violence and economic hardships contribute to depression, anxiety, and conduct problems. These issues are significantly associated with loneliness and sleep impairments, and while our findings do not establish causality, our study highlights the potential occurance of these risks and present a range of cross‑cutting challenges in clinical, psychosocial, adolescent health and educational outcomes.

Previous studies have shown that ACEs contribute to ongoing stress and mental health problems. Our study adds to this evidence on the negative impacts of ACEs on adolescents’ sense of social connectedness and sleep quality, suggesting that in high‑adversity‑exposed adolescents, in addition to addressing ACEs needs, interventions that promote sleep regulation, social connectedness, and self‑regulation might also be needed to more effectively mitigate psychological disturbances for the high‑need population.

Loneliness has been identified in the public mental health field as a silent epidemic. Loneliness works in multiple ways—emanating from sleep impairment and exposure to adversities. Loneliness is on the rise among adolescents, and its insidious impact is commonly seen in depressive disorders [7]. The fact that social functioning and loneliness has an impact on mental disorder development, and social and peer relationships are a critical aspect of development in adolescents, it is critical to strengthen social support intervention to prevent adolescents’ mental health problems. In our final model, we found that sleep impairment is significantly associated with anxiety and anger, but not with depression, when loneliness is also considered. Conversely, loneliness is significantly associated with depression, but not with anxiety or anger. These findings highlight the distinct roles that sleep impairment and loneliness play in different mental health outcomes. Our findings highlight the complex, intertwined relationships among loneliness, sleep impairment, and mental health outcomes in adolescents exposed to high adversity. These factors influence one another in dynamic, potentially bidirectional ways. Loneliness can lead to emotional dysregulation and stress, worsening sleep quality, while sleep disturbances can impair emotional regulation and social engagement, reinforcing loneliness. Both loneliness and sleep impairment are independently associated with increased symptoms of anxiety, depression, and anger, with their co‑occurrence amplifying the issues.

This small study does not represent all adolescents in Nairobi or the wider adolescent populations in Kenya. For advancing the field and promoting contextual, culturally engaged research, longitudinal cohort design that can test a mediational mechanism around ACEs, loneliness, and sleep disturbances would be imperative.

## Conclusion

Our study participants experienced significant psychological disturbances ranging from anxiety, depressive and anger difficulties. Our model suggested that loneliness and sleep impairments are significantly associated with mental health problems in adolescents, even after controlling ACEs. While our cross‑sectional design does not allow for causal inference, these associations highlight the importance of addressing both loneliness and sleep health in mental health interventions. These mechanisms need to be further examined with a larger longitudinal cohort and diverse samples, and with better consideration of contextual and developmental factors that are commonly experienced by adolescents in Kenya and other LMICs.

## Data Accessibility

The datasets generated and analyzed during this study are available from the corresponding author upon reasonable request.

## Role of the Authors

All authors were involved in the planning and execution of the study and had full access to the data. All authors contributed to the manuscript and its review.
